# Access to a main alphaherpesvirus receptor, located basolaterally in the respiratory epithelium, is masked by intercellular junctions

**DOI:** 10.1038/s41598-017-16804-5

**Published:** 2017-11-30

**Authors:** Jolien Van Cleemput, Katrien C. K. Poelaert, Kathlyn Laval, Roger Maes, Gisela S. Hussey, Wim Van den Broeck, Hans J. Nauwynck

**Affiliations:** 1 Department of Virology, Parasitology and Immunology, Faculty of Veterinary Medicine, Ghent University, Salisburylaan 133, 9820 Merelbeke, Belgium; 2 Department of Molecular Biology and Princeton Neuroscience Institute, Princeton University, 119 Lewis Thomas Laboratory, Washington Road, Princeton, New Jersey 08544 USA; 3 Department of Pathobiology and Diagnostic Investigation, College of Veterinary Medicine, Michigan State University, 784 Wilson Road, East Lansing, Michigan 48824 USA; 4 Department of Morphology, Faculty of Veterinary Medicine, Ghent University, Salisburylaan 133, 9820 Merelbeke, Belgium

## Abstract

The respiratory epithelium of humans and animals is frequently exposed to alphaherpesviruses, originating from either external exposure or reactivation from latency. To date, the polarity of alphaherpesvirus infection in the respiratory epithelium and the role of respiratory epithelial integrity herein has not been studied. Equine herpesvirus type 1 (EHV1), a well-known member of the alphaherpesvirus family, was used to infect equine respiratory mucosal explants and primary equine respiratory epithelial cells (EREC), grown at the air-liquid interface. EHV1 binding to and infection of mucosal explants was greatly enhanced upon destruction of the respiratory epithelium integrity with EGTA or N-acetylcysteine. EHV1 preferentially bound to and entered EREC at basolateral cell surfaces. Restriction of infection via apical inoculation was overcome by disruption of intercellular junctions. Finally, basolateral but not apical EHV1 infection of EREC was dependent on cellular N-linked glycans. Overall, our findings demonstrate that integrity of the respiratory epithelium is crucial in the host’s innate defence against primary alphaherpesvirus infections. In addition, by targeting a basolaterally located receptor in the respiratory epithelium, alphaherpesviruses have generated a strategy to efficiently escape from host defence mechanisms during reactivation from latency.

## Introduction

Alphaherpesviruses replicate in the mucosae of the upper respiratory and/or the genital tract of their specific host, which is essential for their efficient transmission. Primary replication in epithelial cells of the mucosa is followed by viral spread in neurons and/or lymphoid tissues and establishment of a lifelong latency^1^. Alphaherpesviruses can reactivate from latency sites when the immune system is compromised, such as during periods of stress. Reactivated virus travels back to respiratory or genital mucosae via anterograde axonal transport or via infected immune cells and efficiently replicates in the epithelium, which results in shedding of infectious virus in respiratory or genital secretions.

The long-term evolutionary relationship between alphaherpesviruses and their hosts has led to the development of several innate mucosal barriers in the latter. These barriers include the mucus layer, the mucociliary escalator, firm intercellular connections and the production of several antimicrobial peptides^2^. Mucus forms the first layer of defence against incoming pathogens. For example in swine, the mucus layer entraps pseudorabies virus (PRV) in the mucoprotein network by charge^3^. In respiratory mucosal explants however, removing the mucus merely increases the number of plaques (unpublished data). We therefore hypothesize that alphaherpesviruses are somehow hindered in their primary replication in the respiratory mucosa by another specific barrier, the intercellular junctions (ICJ). The ciliated pseudo-stratified columnar epithelium has to carefully maintain its integrity and polarity by the action of ICJ. ICJ are specialized regions of contact between adjacent cells and form the morphological and functional barrier between apical and basolateral cell domains. In addition, they ensure resistance against mechanical forces^4^. Tight junctions (TJ) are the most apically located ICJ and function as a size- and ion selective gate for the passage of molecules in between adjacent cells^5,6^. In addition, they prevent diffusion of not only plasma membrane lipids and (glyco)proteins from the apical to the basolateral surface and vice versa, but also of incoming pathogens attached to apical surfaces. Adherent junctions (AJ) are located basally from the TJ and by connecting the cytoskeleton of neighbouring cells, they provide stability and uniformity to the epithelium^7^.

Virus binding and subsequent entry may occur selectively at either the apical or basolateral domains of polarized cells, due to the specific sorting of cell surface receptors. Some viruses (e.g. simian virus 40, respiratory syncytial virus, hepatitis A virus, West Nile virus, chikungunya virus) preferentially infect polarized cells at the apical surfaces, while other viruses (e.g. adenoviruses) prefer basolateral surfaces^8–13^. *In vitro*, herpes simplex virus 1 (HSV1) entry in polarized human uterine (ECC-1), colonic (CaCo-2), and retinal pigment (ARPE-19) epithelial cells occurs most efficiently from the apical surfaces, due to the presence of nectin-1. However, at basolateral surfaces, another putative receptor must function for HSV1 entry, since downregulation of nectin-1 does not influence infection at this site^14^. Moreover, basolateral surfaces of Madin-Darby canine kidney epithelial (MDCK) cells are infected more effectively by HSV1 than their apical surfaces^15,16^. In respiratory epithelial cells, the primary target cells of most alphaherpesviruses, polarity of infection and the importance of ICJ has not been studied to our knowledge. An experimental limitation in previous studies with continuous cell lines is that these do not really reflect the *in vivo* situation. Therefore, we used a respiratory mucosal explant model, which mimics *in vivo* conditions almost perfectly, to investigate the importance of ICJ for the infection of equine herpesvirus type 1 (EHV1), one member of the alphaherpesvirus family. In addition, we isolated primary equine respiratory epithelial cells (EREC) and cultivated them on transwells to examine the polarity of EHV1 binding and subsequent viral replication.

In horses and wild equids, EHV1 infection is widespread and economically very important and induces respiratory disorders, abortion and neurological symptoms^17,18^. The tissue tropism of EHV1 resembles that of the closely related varicella zoster virus (VZV) and HSV1, since these viruses all replicate well in upper respiratory epithelia and to a lesser extent in genital epithelia^19–22^. Horses become infected by EHV1 after uptake of virus-containing secretions, which are transferred between horses through direct or indirect contact^23,24^. In contrast with HSV1, PRV and bovine herpesvirus type 1 (BoHV1), EHV1 is not able to directly cross the basement membrane or to infect respiratory mucosa-associated fibroblasts during primary replication in the respiratory epithelium^20,25–28^. Instead, similar to VZV, EHV1 hijacks leukocytes to cause viremia, spread in the host and establish latency^19,23^. EHV1 infected leukocytes then transfer the virus to local vascular endothelial cells, where viral replication results in vasculitis, thrombosis and edema. In turn, these pathologies lead to severe symptoms such as abortion and nervous system disorders. Viral DNA has also been found in the trigeminal ganglia, suggesting that the virus also establishes latency in neuronal tissues^29,30^. Because vaccines and antivirals are not fully effective, a complete understanding of EHV1 pathogenesis is needed, starting with its primary replication in the respiratory epithelium.

## Materials and Methods

### Virus

Two different Belgian EHV1 isolates were used in this study. The non-neurovirulent strain 97P70 was first isolated in 1997 from the lungs of an aborted foetus^31^. The neurovirulent strain 03P37 originates from the blood taken of a paralytic horse during an outbreak in 2003^32^. Both virus stocks were sequenced in their ORF30 region to confirm the correct genotype and were used at their sixth passage^28^.

### EHV1 purification and Dio-labelling

Virus purification and subsequent Dio-labelling were performed as described previously^33,34^. Full details are given in the supplementary information (SI).

### Tissue collection and processing

The nasal septa and tracheae from different healthy horses were collected at the slaughterhouse and transported in phosphate-buffered saline (PBS) with calcium and magnesium, supplemented with 0.1 mg/mL gentamicin (Invitrogen, Paisley, UK), 0.1 mg/mL kanamycin (Sigma-Aldrich, St. Louis, MO, USA), 100 U/mL penicillin, 0.1 mg/mL streptomycin (Invitrogen) and 0.25 µg/mL amphotericin B (Invitrogen). Nasal septum mucosal explants (nasal ME) and trachea mucosal explants (tracheal ME) were prepared as previously described^27^. Primary equine respiratory epithelial cells (EREC) were isolated and cultured as described by Quintana, *et al*.^35^. Full details are given in the SI.

### Disruption of intercellular junctions

#### Respiratory mucosal explants

Explants were cultured 24 h for adaptation before thoroughly washing and embedding them in agarose diluted in MEM, to mimic *in vivo* conditions, as previously published^36^. Next, the apical surface of the epithelium was exposed for 1 h at 37 °C to 8 mM ethylene glycol tetra-acetic acid (EGTA) (VWR International, Leuven, Belgium), 500 mM N-acetylcysteine (NAC) (Sigma-Aldrich), 20 mM dithiotreitol (DTT) (Sigma-Aldrich) or 50 mM β-mercaptoethanol (Invitrogen) in PBS. As a control, PBS supplemented with calcium and magnesium was used. Explants were removed from the agarose and washed three times in PBS and fixed in a phosphate-buffered 3.5% formaldehyde solution for 24 h, either immediately after the last wash or after an additional 24 h incubation on metal gauzes. Explants were then stored into 70% alcohol until further processing. To guarantee a sufficient viability (>90%) of the explants after treatment with different drugs, an *In Situ* Cell Death Detection Kit (Roche Diagnostics Corporation, Basel, Switzerland), based on terminal deoxynucleotidyl transferase dUTP nick end-labeling (TUNEL), was used. More details are given in the SI.

#### EREC

Cells were grown to confluency and the trans-epithelial electrical resistance (TEER) was measured daily until a steady TEER of ~500–700 Ω∙cm^−2^ was attained. TEER was measured prior to and following treatment with 8 mM EGTA in PBS or, as a control, PBS supplemented with calcium and magnesium. After 30 min, cells were washed three times in PBS. Control cells were incubated an additional 24 h to verify whether damage to the ICJ could be repaired. Viability of the cells was assessed by ethidium monoazide bromide (EMA) staining, ensuring that the treatment with EGTA did not cause a significant cell loss.

### Infection assays

#### Respiratory mucosal explants

Explants were cultured at the air-liquid interface for 24 h, prior to extensive washing and embedment in agarose. Next, explants were treated with EGTA, NAC or PBS to dissociate ICJ, as described above. The apical surface of the epithelium was subsequently inoculated with 10^6.5^ TCID_50_ of the neurovirulent 03P37 strain or the non-neurovirulent 97P70 strain for 1 h at 37 °C. Explants were removed from the agarose and washed 3 times in PBS to remove unbound virus particles. Finally, explants were placed back onto their gauzes and serum-free medium was added. Twenty-four hours post-inoculation, explants were placed in methylcellulose-filled plastic tubes and frozen at −80 °C until further processing.

#### EREC

Apical versus basolateral infection by HSV1 and 2 in a transwell system has already been described by Galen, *et al*.^14^. EREC were grown to full differentiation in a transwell cell culture system and following disruption of ICJ, cells were exposed to 100 µL EHV1 neurovirulent 03P37 or non-neurovirulent 97P70 strain (MOI of 1) at either the apical or the inverted basolateral surface for 1 h at 37 °C. Non-adsorbed virus particles were removed by washing the EREC three times with DMEM/F12. Fresh EREC-medium was added to the platewells and cells were further incubated at the air-liquid interface. Ten hours post inoculation, cells were fixed in methanol for 20 min at −20 °C and stored dry at −20 °C until further processing.

### Binding assays

To characterize the attachment of EHV1 to respiratory mucosal explants and EREC, direct virus-binding studies were carried out with Dio-labelled EHV1-particles. After disruption of the ICJ, cells or explants were chilled on ice for 5 min and washed 3 times with cold PBS. Respiratory mucosal explants were subsequently inoculated with Dio-labelled virus particles at a TCID_50_ of 10^6.5^ for 1 h at 4 °C. EREC were inoculated at a MOI of 10 with Dio-labelled virus particles at either the apical or inverted basolateral surfaces for 1 h at 4 °C. Unbound virus was removed by washing the explants and cells 3 times with cold PBS. Explants were embedded in methylcellulose and frozen at −80 °C until further processing. Cryosections were fixed for 15 min in 4% phosphate buffered paraformaldehyde (PFA) and cells were fixed for 10 min in 1% PFA. Nuclei were counterstained with Hoechst 33342 (10 µg/ml; Invitrogen) for 10 min at room temperature and slides were mounted with glycerol-DABCO. Twenty cryosections of each explant were analysed and the total number of virus particles attached to the apical or basolateral surfaces was counted. The percentage of EREC with bound EHV1 particles was calculated based on the number of cells with viral particles bound on the plasma membrane of 300 randomly selected cells. The number of virus particles attached per cell was calculated based on the number of particles attached at the plasma membrane of 10 random EHV1-positive cells. For each cell, the entire plasma membrane was screened for the presence of virus particles by the use of confocal microscopy.

### Enzymatic removal of cell surface N-linked glycans and sialic acids prior to EHV1 inoculation

To determine the role of different cellular N-linked glycan structures and sialic acids in EHV1 infection of EREC, basolateral and apical cell surfaces were pre-incubated with different enzymes (PNGase F and neuraminidase) or control PBS prior to inoculation with EHV1. More details are given in SI.

### Virus titration

Twenty-four hours after inoculation, explant supernatant was collected and stored at −80 °C until titration. EHV1 titrations were conducted on RK-13 cells, which were incubated at 37 °C for 7 days. Titers were expressed as TCID_50._

### Immunofluorescent staining and confocal microscopy

#### Respiratory mucosal explants

Explants were embedded in methylcellulose and frozen for subsequent cryosectioning. Cryosections were immunofluorescently stained to label late viral glycoproteins, the basement membrane, nuclei, heparan sulfate, chondroitin sulfate or sialic acids. Further details are given in SI.

#### EREC

Immunofluorescent staining to visualize EHV1 immediate early protein (IEP) and cell nuclei was performed directly in the transwells. Further information is given in the SI.

### Statistical analyses

Significant differences (P < 0.05) between different treatments, different virus strains and different inoculation sites were identified by multiple-way analysis of variances (ANOVA) followed by Tukey’s post-hoc test. If homoscedasticity of the variables was not met as assessed by Levene’s test, the data were log-transformed prior to ANOVA. Normality of the residuals was verified by the use of the Shapiro-Wilk test. If the variables remained heteroscedastic or normality was not met after log-transformation, a Kruskall-Wallis’ test, followed by a Mann-Whitley’s post-hoc test were performed. All analyses were conducted in IBM SPSS Statistics for Windows, version 23.0 (IBM Corp, Armonck, NY, USA).

### Data availability statement

The datasets generated during and/or analysed during the current study are available from the corresponding author on reasonable request.

## Results

### EGTA and NAC, but not β-mercaptoethanol nor DTT reversibly disrupt respiratory epithelial intercellular junctions

#### Respiratory mucosal explants

**Figure 1:**
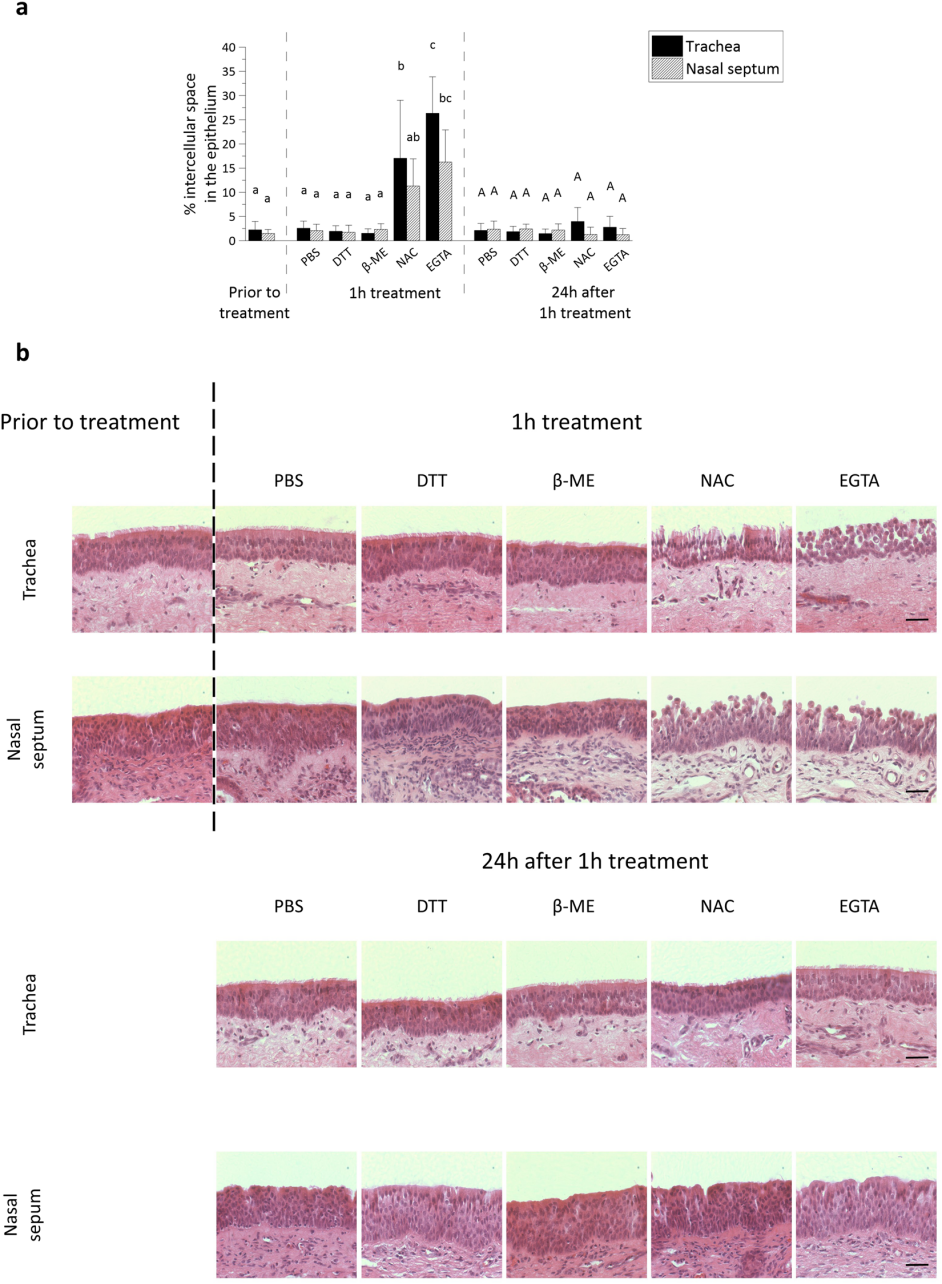
Disruption of ICJ in respiratory mucosal explants. (**a**) The percentage of intercellular spaces in equine respiratory mucosal explants after 1 h treatment with PBS (control), DTT, β-mercaptoethanol, NAC or EGTA (left) and 24 h after the 1 h treatment (right). Three independent experiments were performed and data are represented as means + SD, different lower case letters indicate significant (P < 0.05) differences after 1 h treatment, while different upper case letters indicate significant (P < 0.05) differences 24 h later. (**b**) Representative haematoxylin-eosin stained images of the explants 1 h after treatment (up) and 24 h after the treatment (down). The scale bar represents 50 µm.

As shown in Fig. 1a, the percentage of intercellular spaces in the respiratory epithelium of both nasal and tracheal ME increased after treatment with 8 mM EGTA (16% ± 7% and 26% ± 8%) and 500 mM NAC (11% ± 6% and 17% ± 12%), but not after treatment with 20 mM DTT (2% ± 1% and 2% ± 1%), 50 mM β-mercaptoethanol (2% ± 1% and 2% ± 1%) or control PBS (2% ± 1% and 2% ± 1%). Representative haematoxylin-eosin images are shown in Fig. 1b. The effect of NAC on intercellular bridges disappeared after decreasing its concentration ten-fold. Increasing the concentration of DTT or β-mercaptoethanol by ten-fold did not alter the intercellular spaces, but did decrease cell viability, as assessed with TUNEL-staining (data not shown). Cell viability in the respiratory mucosal explants did not significantly drop after treatment with 8 mM EGTA or 500 mM NAC, compared to control PBS (SI Fig. S1). Notably, the intercellular spaces are wider in tracheal ME, when compared to nasal ME after disruption with NAC and EGTA. Twenty-four hours after the 1 h treatment, samples were analysed to determine whether the respiratory epithelium was able to repair its ICJ. Indeed, the percentage of intercellular spaces in the respiratory epithelium of tracheal ME decreased back to 4% ± 3% 24 h after the 1 h treatment with NAC and to 3% ± 2% 24 h after the 1 h treatment with EGTA (Fig. 1a). In nasal ME, a similar decrease in percentage of intercellular spaces was observed 24 h after the 1 h treatment with NAC and EGTA (2% ± 1% and 2% ± 1%, respectively).

#### EREC

EREC attained a steady trans-epithelial electrical resistance (TEER) of ~500–700 Ω∙cm^−2^ after 5–7 days of incubation at the air-liquid interface in a transwell cell culture system. The TEER significantly dropped to baseline levels after treatment with EGTA, but not after treatment with PBS (SI Fig. S2a). EGTA did not alter cell viability, as determined by EMA-staining (SI Fig. S2b). Twenty-four hours after treatment, EREC regained a stable TEER of 500–700 Ω∙cm^−2^, indicating that EREC are able to restore their intercellular bridges efficiently.

### Intercellular junctions protect respiratory mucosal explants from EHV1 infection

#### Number of plaques

**Figure 2:**
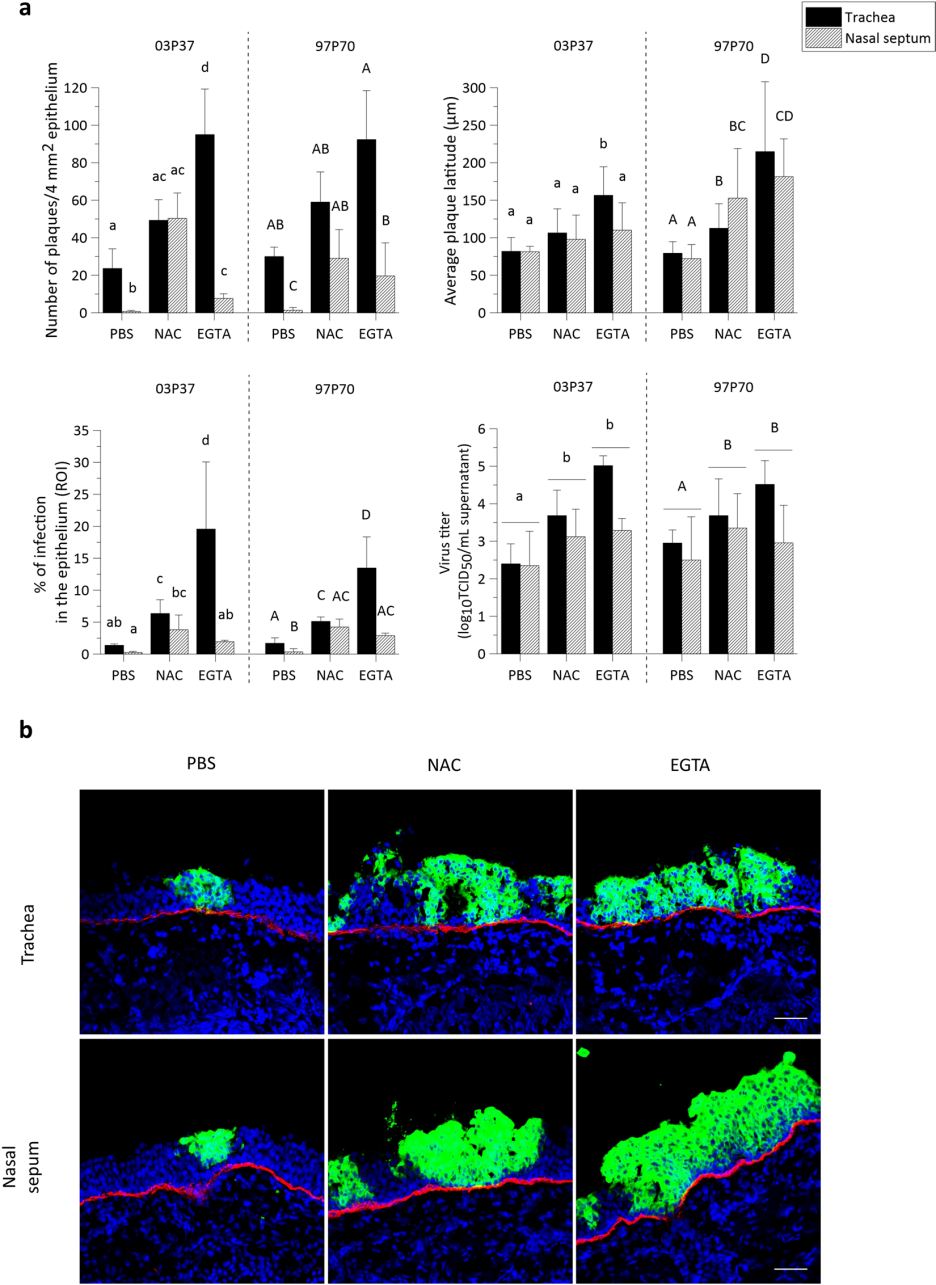
EHV1 infection of respiratory mucosal explants after disruption of ICJ with NAC or EGTA. (**a**) Respiratory mucosal explants were pre-incubated with NAC, EGTA or control PBS, prior to inoculation with EHV1 03P37 or 97P70 strain for 1 h at 37 °C (10^6.5^ TCID_50_). Explants were frozen 24hpi and cryosections were stained for late viral antigens. The total number of plaques was counted on 50 consecutive cryosections, the average plaque latitude was calculated based on a maximum of 10 individual plaques, the percentage of EHV1 infection in the epithelium was analysed on 5 random cryosections and the virus titer was determined in supernatant on RK-13 cells. Data are represented as means + SD and different lower case numbers indicate significant (P < 0.05) differences in 03P37 strain infection, different upper case letters represent significant (P < 0.05) differences in 97P70 strain infection. Experiments were performed on 3 individual horses. (**b**) Representative confocal images of EHV1 plaques (green) in respiratory mucosal explants. The basement membrane is shown in red. Cell nuclei were counterstained with Hoechst (blue). The scale bar represents 50 µm.

As shown in Fig. 2a, upper left panel, the number of plaques per 50 cryosections increased from 23 ± 10 (03P37) and 30 ± 5 (97P70) in control treated tracheal ME to 49 ± 11 (03P37) and 59 ± 16 (97P70) in NAC treated tracheal ME and to 95 ± 24 (03P37) and 92 ± 26 (97P70) in EGTA treated tracheal ME. Similarly, the number of plaques was significantly higher in nasal ME after NAC (50 ± 14 for 03P37 and 29 ± 15 for 97P70) and EGTA (8 ± 3 for 03P37 and 19 ± 17 for 97P70) pre-treatment, compared to control pre-treatment (1 ± 1 03P37 and 1 ± 1 97P70). In control treated explants, the number of plaques in nasal ME was significantly lower compared to tracheal ME. After disruption of the ICJ by NAC however, the number of plaques in nasal ME did no longer significantly differ from NAC pre-treated tracheal ME. Remarkably, treatment with EGTA did not completely overcome this restriction in nasal ME.

#### Plaque latitude

The plaque latitude gives an indication about the ease of viral spread in the explant and is shown in Fig. 2a, upper right panel. The average latitude of 03P37 strain plaques increased from 82 ± 18 µm in control treated tracheal ME to 106 ± 32 µm and 157 ± 38 µm after NAC and EGTA treatment, respectively. Similarly, the average latitude of the 97P70 strain plaques was significantly higher after NAC and EGTA treatment (113 ± 33 µm and 215 ± 93 µm respectively), compared to control (79 ± 15 µm). A similar pattern was observed in nasal ME.

#### Percentage of infection in the epithelium

In order to obtain a general view of EHV1 infection in explants, the percentage of infection in the epithelium (i.e. ROI) was calculated and is illustrated in Fig. 2a, lower left panel. In control treated tracheal ME, 1.38% ± 0.21% of the epithelium was infected by the 03P37 strain and 1.71% ± 0.83% by the 97P70 strain. Pre-treatment of tracheal ME with NAC led to a significant increase of this percentage to 6.35% ± 2.15% (03P37) and 5.12% ± 0.69% (97P70) 24hpi. Up to 19.57% ± 10.49% and 13.48% ± 4.86% of the epithelium got infected by the 03P37 strain and the 97P70 strain, respectively, after pre-treatment with EGTA. A similar trend, although with lower values, was observed in nasal ME. In control treated explants, only 0.23% ± 0.17% was infected with the 03P37 strain and 0.35% ± 0.48% with the 97P70 strain. This percentage increased significantly to 3.81 ± 2.29 (03P37), 4.24 ± 1.24 (97P70) and 1.93% ± 0.24% (03P37), 2.88% ± 0.4% (97P70) after NAC and EGTA treatment, respectively.

#### Virus titer

More efficient virus replication in the epithelium most likely results in the production of more extracellular virus particles. Indeed, virus titration on RK13 cells (Fig. 2a, lower right panel) confirmed this hypothesis showing that EGTA treated tracheal ME supernatant contained a 2 to 3 log higher (03P37) and 1 to 2 log higher (97P70) EHV1 titer than control treated tracheal ME supernatant. NAC treated tracheal ME supernatant was on average 1 log (03P37 and 97P70) higher than control supernatant. In general, the 03P37 and 97P70 titers of nasal ME supernatant were 0.5 to 1.5 log higher after treatment with NAC or EGTA, compared to controls.

Representative confocal images of EHV1 plaques in the respiratory mucosal explants are shown in Fig. 2b.

### EHV1 preferentially infects basolateral surfaces of respiratory epithelial cells

#### Number of plaques

**Figure 3:**
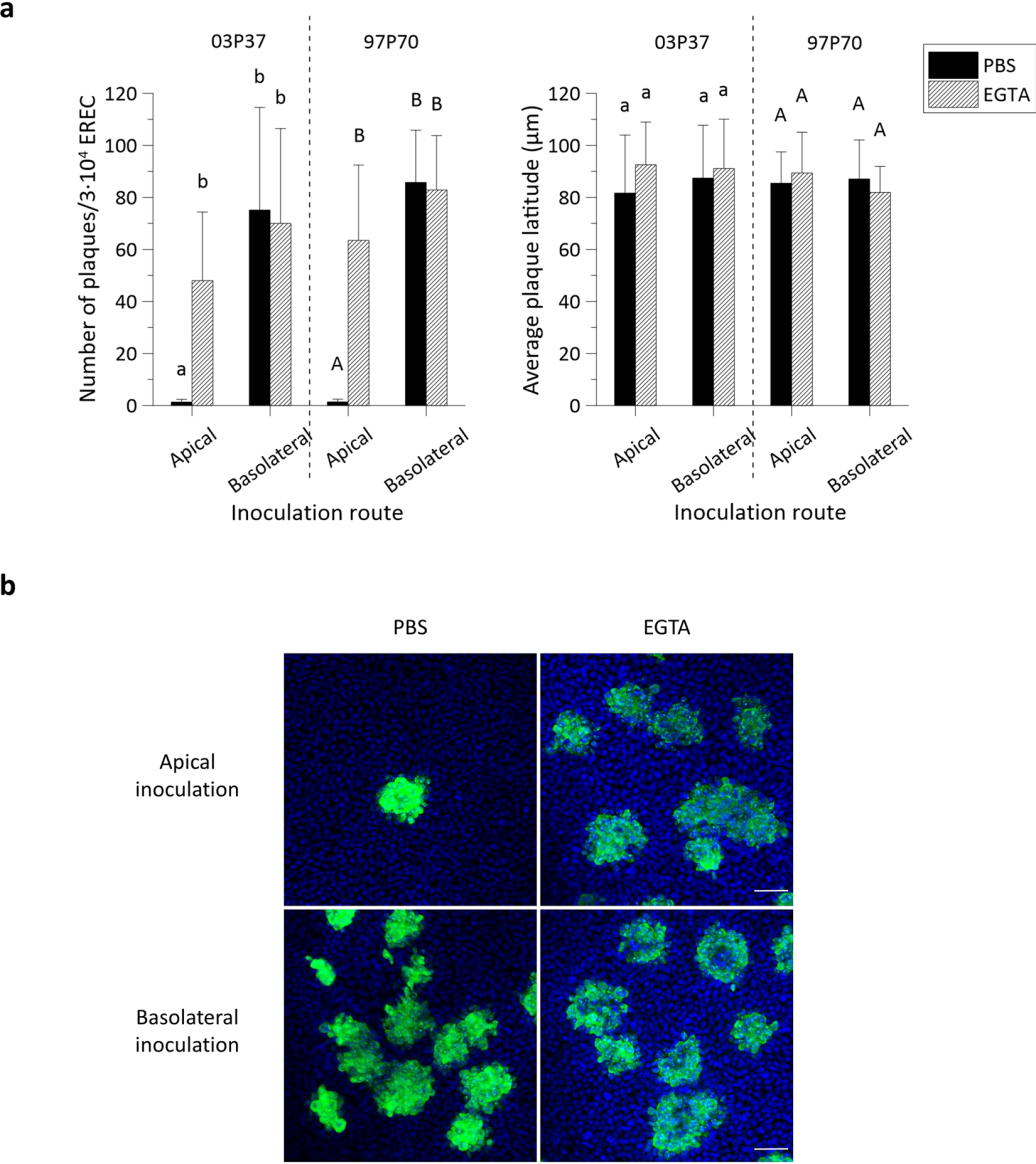
EHV1 preferentially infects the basolateral surface of EREC and disruption of ICJ overcomes the restriction to EHV1 infection at the apical surface. (**a**) To compare EREC susceptibility to EHV1, cells were exposed at either the apical surface or basolateral surface to EHV1 (MOI 1). Cells were fixed in methanol 10 hpi and stained for IEP. The total number of plaques was counted in five different fields of approximately 3∙10^4^ cells for each condition (left). Average plaque latitudes were measured on 10 individual plaques (right). Experiments were performed in triplicate on primary EREC of 3 different horses. Data are represented as means + SD and significant (P < 0.05) differences for 03P37 strain infection are indicated by lower case letters and for the 97P70 strain infection by upper case letters. (**b**) Representative confocal images of EHV1 IEP-positive plaques (green) in EREC monolayers, nuclei were detected with Hoechst (blue). The scale bar represents 50 µm.

To investigate whether EHV1 preferentially infects either apical or basolateral surfaces of EREC, we inoculated the cells by either route. On 3∙10^4^ EREC, we counted an average of 1 ± 1 viral plaques after apical inoculation with both the 03P37 and the 97P70 strain. Basolateral inoculation with the respective strains led to a 58 ± 30-fold and 61 ± 14-fold increase in the number of plaques on an area of 3∙10^4^ EREC, as shown in Fig. 3a, left panel.

To determine whether ICJ also protect the basolateral surfaces of primary isolated EREC from EHV1 infection, cells were treated with 8 mM EGTA before inoculation at the apical or inverted basolateral surfaces. Disruption of the ICJ increased EREC infection by 37 ± 20-fold and 45 ± 21-fold after inoculation at the apical surface with the 03P37 and 97P70 strain, respectively. Treatment with EGTA did not significantly change infection after inoculation at the basolateral surfaces with neither of the 2 strains.

#### Plaque latitude

No significant difference in EHV1 plaque latitude was found between different inoculation routes or between different treatments (Fig. 3a, right panel).

Representative confocal images are shown in Fig. 3b. Since no significant differences were observed between the two different strains, all further experiments were conducted with the 03P37 strain only.

### Increased epithelial cell susceptibility to EHV1 at the basolateral surfaces is correlated with the virus binding step

Here, we examined EHV1 binding to respiratory mucosal explants after control PBS, EGTA or NAC treatment and to EREC after control PBS or EGTA treatment.

#### Respiratory mucosal explants

The 03P37 strain was purified and Dio-labelled as previously described^33^. In PBS treated tracheal ME and nasal ME, a respective total number of 320 ± 28 and 179 ± 61 virus particles was counted on the plasma membrane. This difference shows that the limited infection of nasal ME, compared to tracheal ME, can in part be related to a reduced viral attachment.

**Figure 4:**
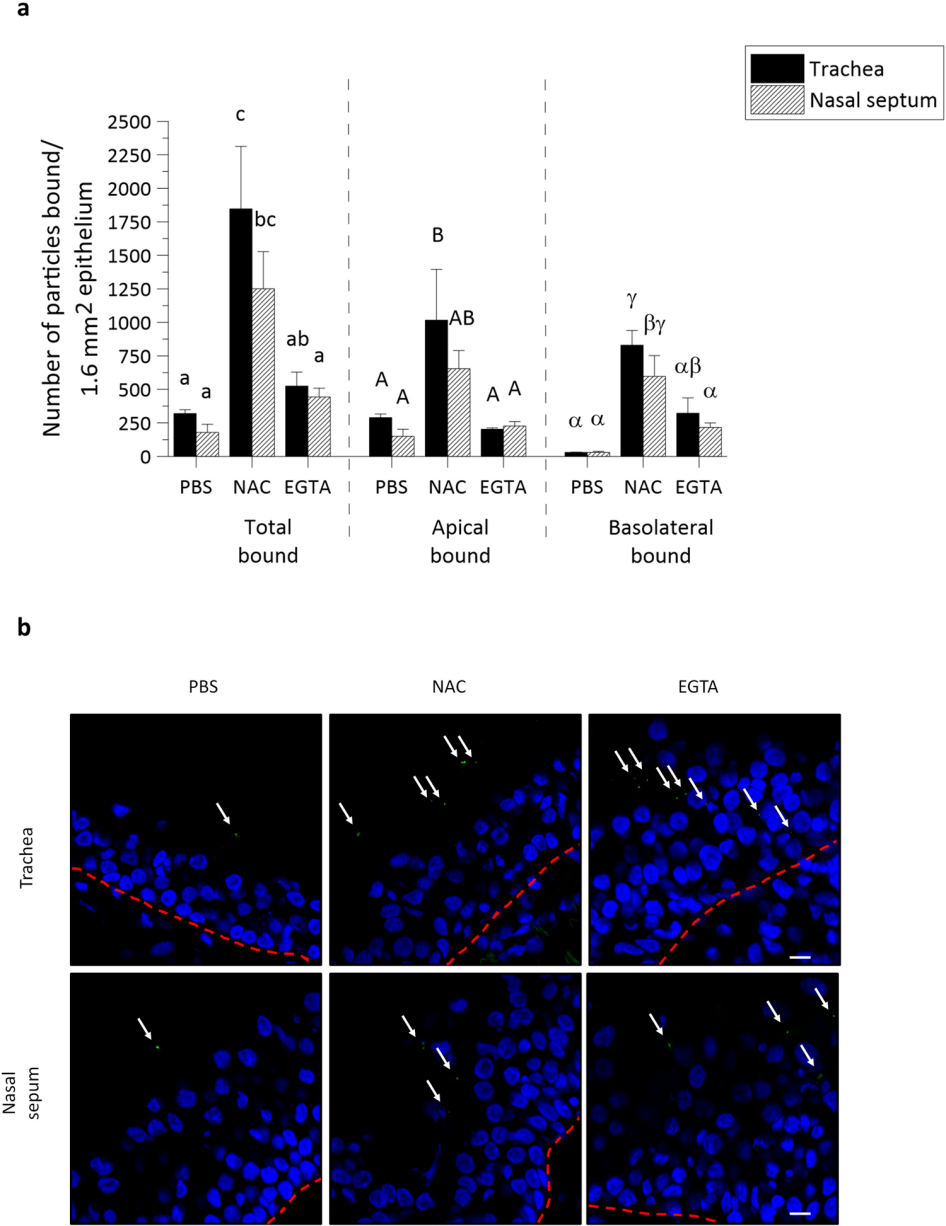
EHV1 attachment to equine respiratory mucosal explants is enhanced after disruption of tight junctions with NAC and EGTA. (**a**) Dio-labelled EHV1 particles (10^6.5^ TCID_50_) were added for 1 h at 4 °C to the pre-treated tracheal ME and nasal ME (PBS, NAC or EGTA). The total number, the number bound to the apical surfaces and to the basolateral surfaces were individually counted on 20 cryosections of 16 µm. Three independent experiments were performed and data are represented as means + SD, different lower case letters represent significant (P < 0.05) differences in the total number of attached EHV1 particles. Significant (P < 0.05) differences in the number of EHV1 particles bound to the apical surfaces of explants are indicated by upper case letters. Different Greek letters indicate significant (P < 0.05) differences in the number of EHV1 particles bound to the basolateral surfaces of explants. (**b**) Representative confocal images of Dio-labelled EHV1 particles attached to tracheal ME and nasal ME upon different treatments (PBS, NAC or EGTA). The white arrows point at virus particles and the red dotted line represents the basement membrane. The scale bar measures 10 µm.

To more specifically evaluate the area of virus attachment, virus particles attached to either the apical surface or the basolateral surface were separately counted. As shown in Fig. 4a, the difference in virus binding between both types of tissue was only found at the apical surface and not at the basolateral surfaces. Upon PBS treatment, 289 ± 28 EHV1 particles bound to the apical surface of tracheal ME, compared to 150 ± 52 EHV1 particles at the apical surface of nasal ME, whereas only 30 ± 1 and 30 ± 8 EHV1 particles were found at the basolateral surfaces of the respective mucosal explants.

Treatment with EGTA and subsequent disruption of ICJ integrity did not significantly increase the binding of EHV1 to the apical surfaces of both tracheal ME and nasal ME (203 ± 33 and 227 ± 32, respectively). However, EHV1 binding to the basolateral surfaces increased to 322 ± 114 in tracheal ME and 216 ± 33 in nasal ME. Treatment of the explants with NAC, which was shown above to damage intercellular bridges and which is also being used as a mucolytic drug, did increase the number of viral particles attached to both the apical and basolateral surfaces of tracheal ME (1017 ± 378 and 830 ± 109) and nasal ME (654 ± 136 and 659 ± 155).

Representative confocal images are shown in Fig. 4b.

#### EREC

**Figure 5:**
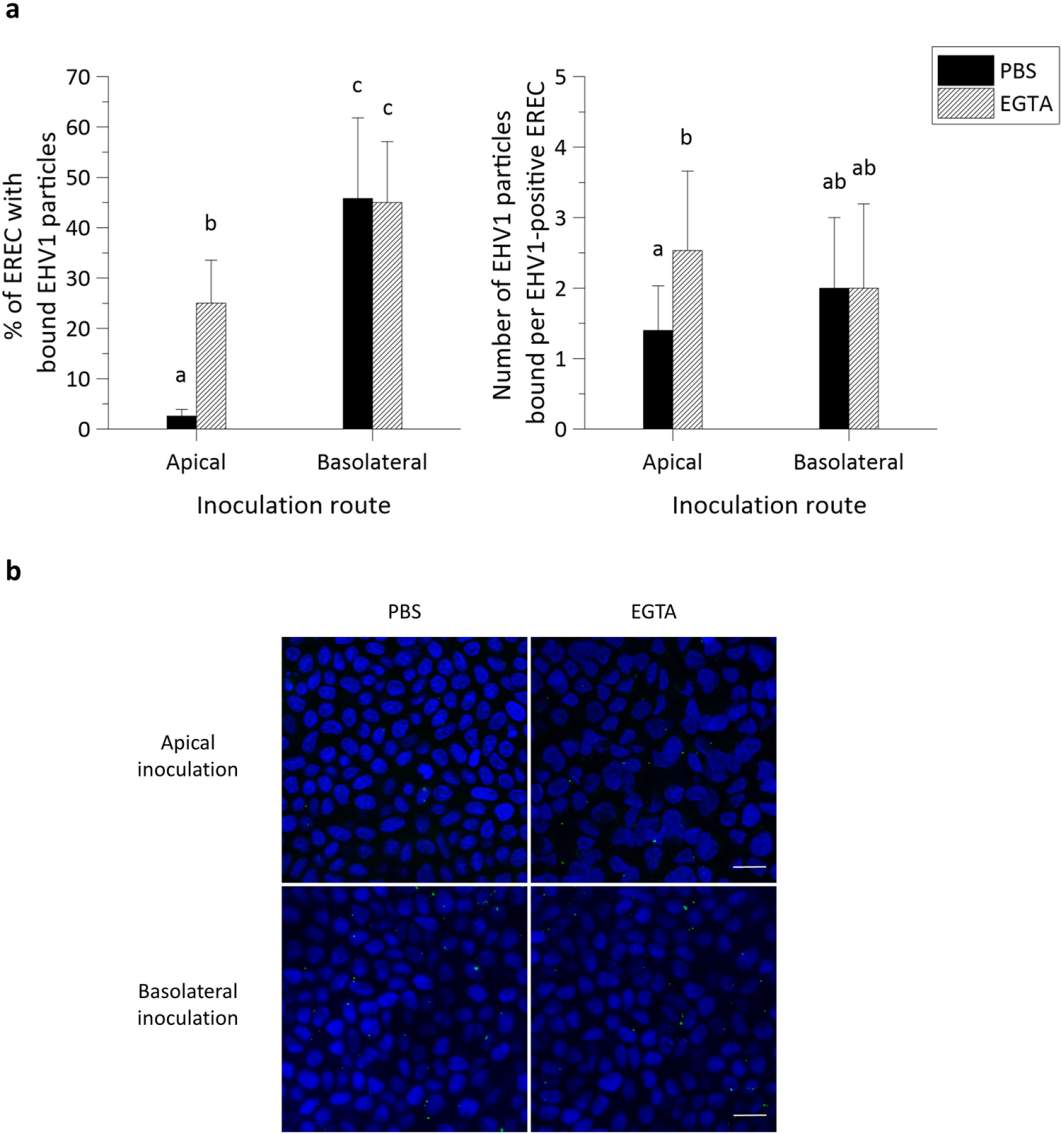
EHV1 binds up to 10-fold better at basolateral surfaces than at apical surfaces of EREC with intact intercellular bridges. (**a**) Cells were pre-incubated with PBS or EGTA and inoculated at 4 °C for 1 h with Dio-labelled EHV1 particles (MOI 10). The percentage of cells with bound virus particles was calculated based on 5 random fields of 300 cells (left panel). The total number of particles per EHV1-positive cell was counted (right panel). Three independent experiments were performed and data are represented as means + SD, different letters indicate significant (P < 0.05) differences. (**b**) Representative confocal images of EREC with bound EHV1 particles (green), cell nuclei are stained in blue, the scale bar represents 10 µm.

These findings in explants were corroborated in EREC from three individual horses. As represented in Fig. 5a, left panel, the percentage of EREC with bound EHV1 particles was significantly higher (46% ± 16%) after inoculation at the basolateral surfaces, compared to apical inoculation (3% ± 1%). Disruption of ICJ had a similar effect on EHV1 binding to EREC as in explants, where 25% ± 8% of the cells had virus particles attached to their plasma membrane upon apical inoculation. Intercellular junction disruption in EREC had no effect on EHV1 binding upon basolateral inoculation (45% ± 9%), compared to control EREC with intact ICJ.

The number of virus particles bound to the plasma membrane of EHV1-positive EREC significantly increased after treatment with EGTA (3 ± 1), compared to PBS treatment (1 ± 1), upon apical inoculation (Fig. 5a, right panel). Upon basolateral inoculation, the number of virus particles per EHV1-positive cell in either PBS or EGTA treated EREC was similar (2 ± 1 and 2 ± 1, respectively). These results are visualized with confocal images in Fig. 5b.

### N-glycans, but not heparan sulfate, chondroitin sulfate or sialic acids play a role in EHV1 infection of respiratory epithelial cells

#### Expression pattern in the respiratory epithelium

**Figure 6:**
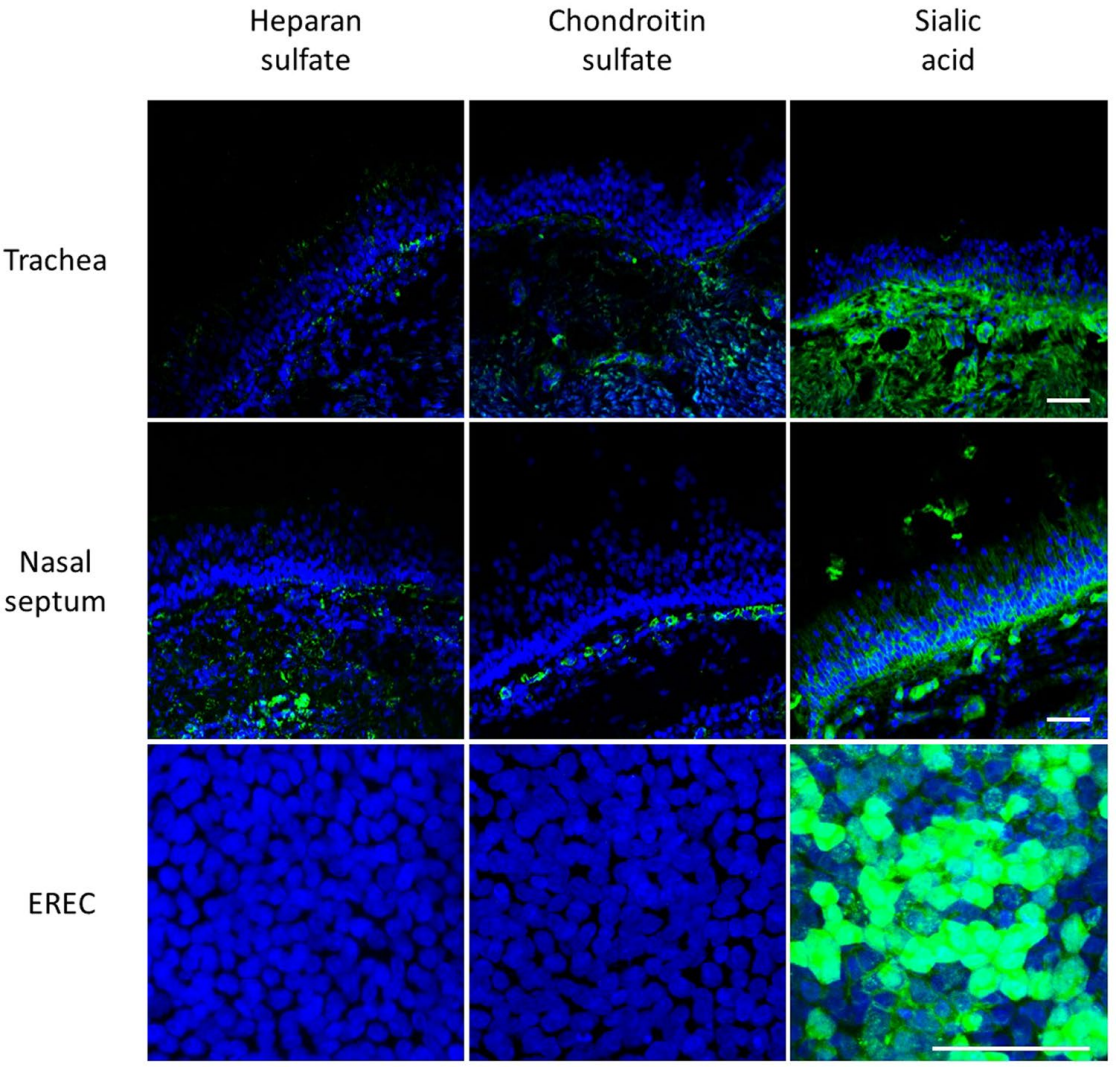
Localisation of heparan sulfate, chondroitin sulfate and α2,3-linked sialic acid expression by confocal microscopy. Cryosections and cells were fixed in PFA before permeabilisation with Triton X. Heparan sulfate and chondroitin sulfate were visualized by labelling with monoclonal antibodies 10E4 and CS-56 (green), α2,3-linked sialic acids were detected by biotinylated Maackia Amurensins lectin (green). Hoechst stained cell nuclei blue. Respiratory mucosal explants express heparan sulfate and chondroitin sulfate at the basolateral membrane and α2,3-linked sialic acids all over the plasma membrane (green) (upper and middle panel). Isolated EREC monolayers solely express α2,3-linked sialic acids and not heparan sulfate, nor chondroitin sulfate (lower panel). The scale bars represent 50 µm.

Cellular glycosaminoglycans and sialic acids are known to play a role in the attachment of several herpesviruses, including HSV1, PRV, BoHV1 and EHV1^33,37–39^. The presence of these cellular glycosaminoglycans and sialic acids was first verified by immunofluorescent staining of respiratory mucosal explant cryosections. As shown in Fig. 6 left panel, heparan sulfate is present solely at the basal site of the equine respiratory epithelium. A similar pattern was observed for the distribution of chondroitin sulfate in equine respiratory epithelium (Fig. 6 middle panel). The lectin Maackia Amurensis binds α2,3-linked sialic acids with high affinity, and not α2,6-linked sialic acids. Sialic acids are distributed equally all over the plasma membrane of epithelial cells in respiratory mucosal explants, as shown in Fig. 6, right panel. Isolation of primary cells might affect their glycosaminoglycan expression pattern and indeed, immunofluorescent staining of EREC turned out negative for heparan sulfate and chondroitin sulfate, but remained positive for sialic acids.

#### Role in EHV1 infection

**Figure 7:**
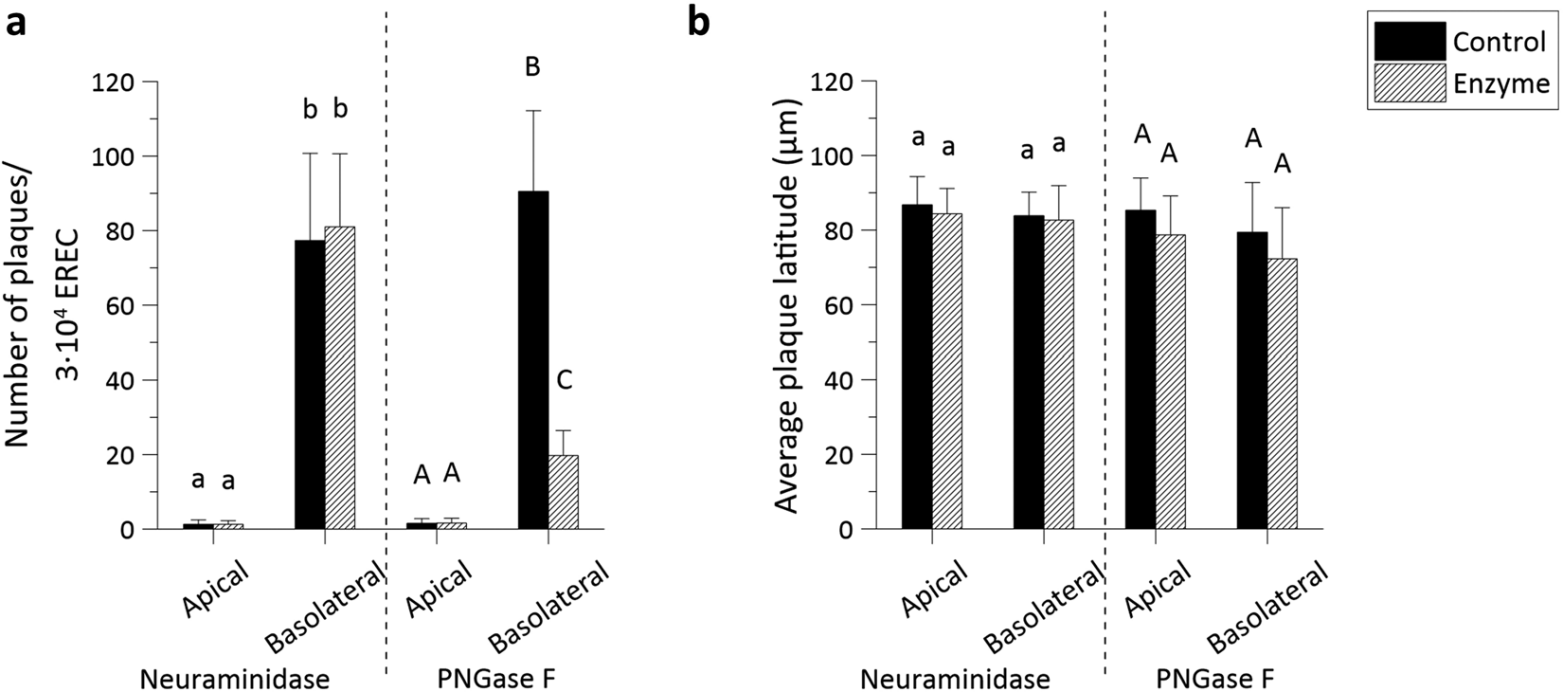
N-linked glycans, but not sialic acids play a role in EHV1 infection of EREC after basolateral inoculation. EREC were grown to confluency on transwells before enzymatic treatment with neuraminidase (1 h, 37 °C) or PNGase F (12 h, 37 °C) at either the apical or basolateral surface. Next, the same route was used for inoculation with EHV1 (MOI 1, 1 h, 37 °C) and cells were fixed in methanol 10 hpi before IEP staining. The total number of plaques was counted in five different fields of approximately 3∙10^4^ cells for each condition (left). Average plaque latitudes were measured on 10 individual plaques (right). Experiments were performed in triplicate on primary EREC of 3 different horses. Data are represented as means + SD and significant (P < 0.05) differences are indicated by different letters.

To explore whether these sialic acids or, more generally, N-linked glycans, are involved in EHV1 infection, sialic acids or N-linked glycans were enzymatically cleaved by neuraminidase or PNGase F, respectively, from either the apical or basolateral cell surface prior to inoculation with EHV1 at the respective routes. As a positive control, MDCK cells were inoculated by an A/equine/Kentucky/98 influenza strain (H3N8) after neuraminidase treatment. Basolateral enzymatic removal of N-linked glycans rendered EREC 4-fold less susceptible to EHV1, compared to the control, as determined by counting the number of plaques on 3∙10^4^ EREC (Fig. 7a). No significant difference in number of plaques could be detected between control PBS treated cells and neuraminidase treated cells, while equine influenza virus (EIV) infection of MDCK cells was significantly lower after enzymatic removal of sialic acids by neuraminidase (SI Fig. 3a). To further verify the correct cleavage of sialic acids on EREC surfaces, immunofluorescent staining was performed to compare the relative amount of sialic acids before and after enzymatic treatment by measuring the intensity of the fluorescent signal on 10 different z-stacks. The enzyme could correctly remove sialic acids on either the apical or basolateral cell surfaces (SI Fig. 3b). Finally, plaque latitude did not significantly differ among different enzymatic treatments (Fig. 7b). These findings demonstrate that EHV1 uses cellular N-linked glycans, but not heparan sulfate, nor chondroitin sulfate, nor sialic acids for initial infection of EREC.

## Discussion

The respiratory mucosa plays a vital role in the transmission of alphaherpesviruses. These viruses not only enter their host through infection of the respiratory tract, they also use the respiratory system to spread to new hosts later on in their life-cycle. Until now, the specific relationship between the respiratory mucosa integrity and polarity on one hand and alphaherpesvirus infection on the other hand has not been studied. Previous studies examining polarity of alphaherpesvirus infections have been conducted in continuous cell lines, different from the *in vivo* replication sites. Here, we use an established equine respiratory mucosal explant model and to our knowledge, this study is the first to disrupt intercellular bridges in a mucosal explant system. In cell cultures, measuring the trans-epithelial electrical resistance or the leakage of labelled dextran or albumin across the apical and basolateral compartments of transwells is the standard method to verify epithelial permeability^14,40–43^. *In vivo*, epithelial integrity could be assessed by measuring the trans-epithelial electrical voltage with an electrode, placed in the trachea^41^. However, none of these methods could be applied on mucosal explants and therefore, we set up a new protocol to verify ICJ integrity by examining the intercellular space in haematoxylin-eosin stained paraffin coupes by means of image analysis. Equine tracheal and nasal ME were incubated with various metal chelating and/or reducing drugs in order to disrupt epithelial integrity. Only the drugs affecting extra- and intracellular calcium levels (EGTA and NAC), and not the reducing agents affecting disulfide bonds in adhesion proteins (DTT or β-mercaptoethanol), were able to alter nasal and tracheal epithelial integrity. Calcium is the most important ion in regulating intercellular junction stability. Homophilic interactions between AJ directly depend on extracellular calcium availability^7^. Alteration of intracellular calcium concentrations or depletion of extracellular calcium also mediates the redistribution of AJ and TJ proteins^44,45^. EGTA specifically sequestrates extracellular calcium and adding it to cell culture medium of polarized epithelial cells rapidly results in splitting of their ICJ^46^. NAC modulates intracellular calcium levels but until present, it was not proven to alter integrity of ICJ^47^. Of these two drugs, EGTA most efficiently destroyed the ICJ, possibly due to its direct action in the destruction of ICJ protein interactions, compared to NAC, which indirectly modulates ICJ proteins. Notably, a rather high concentration of NAC was necessary to disrupt intercellular integrity in explants, which was toxic when applied directly on EREC. These findings point out that care must be taken when using the mucolytic drug Lysomucil^®^ to treat horses with chronic obstructive pulmonary disease or with severe pneumonia^48,49^.

Although not significantly, destruction of epithelial integrity by both EGTA and NAC occurs more efficiently in tracheal ME, compared to nasal ME. Our observations complement those of previous *in vitro* and *ex vivo* studies, showing that the average trans-epithelial electrical resistance and subsequent ICJ integrity progressively decreases from proximal to distal airways^50–52^. The resistance against incoming pathogens is also obvious after challenging the nasal septum with EHV1. In control treated explants, EHV1 infection is significantly lower in nasal ME than in tracheal ME. This limited infection is presumably a direct consequence of impaired virus binding, considering the low number of EHV1 particles attached to nasal ME. As the primary air filter, the nasal septum is guarded with an overlying mucoprotein network, a specialized glycocalix, a repertoire of antimicrobial peptides and firm intercellular contacts, which might entrap and neutralize incoming virus particles more efficiently^53^. Disruption of ICJ with EGTA did not completely overcome the restriction in EHV1 infection of nasal ME. However, after pre-treatment with NAC, infection was greatly enhanced to a similar level as in tracheal ME. It is known that NAC acts as a mucolytic by disrupting disulfide bonds in the mucoprotein network^54^. Remarkably, using DTT and β-mercaptoethanol prior to EHV1 inoculation did not increase subsequent infection (data not shown). Thus in nasal ME, a similar destruction of the mucoprotein network and of ICJ is necessary in order for EHV1 to efficiently infect the epithelial cells. In addition, we observed a significant increase in EHV1 binding to apical surfaces of nasal and tracheal ME after NAC pre-treatment, pointing out the relevance of the overlying mucoprotein network in both tissues. Disruption of intercellular contacts also results in greater virus binding to basolateral surfaces of the explants. First of all, virus particles are able to migrate in between neighbouring cells and get in direct contact with basolateral surfaces. Secondly, virus particles attached to transmembrane proteins in apical surfaces can freely move to the basolateral side of the plasma membrane, since cell polarity is lost^55^.

The results obtained with *ex vivo* respiratory mucosal explants most likely reflect what happens *in vivo* when epithelial integrity is lost. It has been shown that ICJ integrity is affected by several factors including pollens, mycotoxins, bacterial toxins, inflammatory cytokines (INF-γ, TNF-α and interleukins) and hormones (estradiol)^42,56–61^. Pollens exhibit proteolytic activities for efficient pollination, but when inhaled by humans or animals, these proteases can destruct TJ proteins^42,58^. Allergic reactions to pollens are linked to asthma and in humans, it is known that allergic patients tend to have a less firm epithelial barrier^62^. Chronic allergies in pasture-kept horses are known as ‘summer pasture recurrent airway obstruction’ (SP-RAO) and are associated with inhaling pollens, moulds and mycotoxins^63^. It can be postulated that as a consequence, horses affected with SP-RAO might have a deficiency in epithelial integrity and therefore be more receptive for an EHV1 infection and shed EHV1 more easily towards other horses. Chronic airway inflammation is also linked to chronic microbial infections. Bacterial toxins and cellular cytokines produced upon viral and/or bacterial infections can directly alter ICJ protein redistribution and induce the development of asthma^60,61^. Hormones such as estrogens are important for the regulation of the estrous cycle and the maintenance of pregnancy. In order to modulate its vascular functions (i.e. angiogenesis), estrogens need to disrupt cell-cell adhesions to allow the migration of endothelial cells^59^. In horses, fetoplacental estrogens slowly increase during pregnancy and blood concentrations peak at late term gestation. Remarkably, horses are most susceptible to EHV1 abortion during this period^17^. Smith, *et al*.^64^ showed that 17β-oestradiol activates the expression of adhesion molecules on endothelial cells of the reproductive tract, which is a key step in transferring virus from infected leukocytes to endothelial cells. The precise role of 17β-oestradiol on the integrity of equine endothelial cells and subsequent transfer of EHV1 has not been studied. Destruction of endothelial integrity might facilitate the spread of EHV1 and furthermore, mares might be more sensitive for a respiratory re-infection when the intercellular bridges at the respiratory tract are damaged by estrogens. Indeed, it has been shown that the nasal respiratory epithelium is an estrogen target^65^.

Since disruption of respiratory epithelium integrity leads to enhanced EHV1 binding, we hypothesized that its primary binding/entry receptor is located basolaterally. Therefore, we isolated EREC and inoculated them at either apical or basolateral surfaces. Indeed, we found that EHV1 preferentially binds to and infects EREC at basolateral surfaces. Along with EHV1, more respiratory viruses target a basolaterally located receptor, which is only exposed in a compromised epithelium. Adenoviruses bind to an integral TJ protein named ‘coxsackievirus and adenovirus receptor’, which is only accessible after disruption of epithelial integrity^66^. HSV1 most efficiently infects human epithelial cells from the apical surface, due to the presence of nectin-1. However, downregulation of nectin-1 had no impact on basolateral infection, indicating that HSV1 can also use this putative basolaterally located receptor^14^.

It is known that EHV1 initially interacts with heparan sulfate on cell surfaces via gB and gC^37,67^. The respiratory epithelium expressed heparan sulfate solely at the level of the basement membrane. This distribution pattern is in accordance with MDCK- and murine mammary epithelial cells, which preferentially sort heparan sulfate to their basolateral domain^68,69^. However, primary isolated EREC lacked expression of heparan sulfate and in addition, pre-treatment of EHV1 with heparin did not reduce subsequent EREC infection (unpublished data). Similar to heparan sulfate, chondroitin sulfate was present in the basolateral membrane of nasal and tracheal ME, but was absent in EREC. As assessed by immunofluorescent staining, sialic acids were ubiquitously expressed on EREC. Moreover, sialic acids are shown to play a role in EHV1 entry in monocytic CD172a^+^ cells^33^. Therefore, we specifically removed sialic acids with neuraminidase prior to EHV1 inoculation. However, no significant reduction in number of virus plaques was observed between neuraminidase and control treated EREC. These results indicate that other glycosaminoglycans play a role in EHV1 infection of EREC. Indeed, after non-specifically removing cellular N-linked glycans, subsequent EHV1 infection was impaired. Next, EHV1 glycoprotein gD binds to a cellular receptor to stabilise the binding and trigger entry into the cell^70^. MHCI is shown to be a putative receptor for EHV1 gD in equine brain microvascular cells, dermal cells and PBMC^71,72^. However, MHCI is ubiquitously expressed and therefore, does not correlate with EHV1 tissue tropism. EHV1 entry in PBMC is initiated by binding of gD to cellular integrins^33,73^. Integrins such as α3β1, α6β4 and αvβ5 are sorted to the basolateral domains of respiratory epithelial cells, allowing firm attachment to the extracellular matrix^74^. The importance of these integrins in EHV1 infection of EREC is still unknown. Finally, nectins aid in calcium-independent cellular adhesion and are known to interact with several alphaherpesviruses, including HSV1 and 2, PRV and BoHV1^75^. Nonetheless, EHV1 presumably does not depend on nectins, since CHO-K1 lack nectin-1 and 2 expression, although they are fully susceptible to EHV1^76^. Until present, the precise EREC binding/entry receptor for EHV1 remains unknown. Our observations point out that it is located at cellular basolateral surfaces and becomes apically accessible when intercellular adhesion is impaired.

We hypothesize that EHV1, among other alphaherpesviruses, exhibits a specific strategy for host invasion and subsequent spread. Upon inhalation, primary virus replication in respiratory epithelial cells is limited in order to avoid the onset of a strong immune response, but high enough to infect latency inducible cells (leukocytes and neurons). Once present inside the host, the virus can invade the respiratory epithelium from its basolateral side upon reactivation. Spread can occur from infected leukocytes or from infected neurons through anterograde axonal transport. This is key for efficient secondary replication in the respiratory epithelium and shed of progeny virus to new hosts.

Taken together, our results shed new light on the pathogenesis of alphaherpesviruses in their specific target host cells, a significant aspect of medical research on herpesvirus-induced diseases.

## Electronic supplementary material

Supplementary information
