# Supplementary material for: Access to a main alphaherpesvirus receptor, located basolaterally in the respiratory epithelium, is masked by intercellular junctions
Source: Sci Rep. 2017 Nov 30;7:16656. doi: 10.1038/s41598-017-16804-5 (PMC5709510; doi:10.1038/s41598-017-16804-5)
Supplement: Supplementary file 1 — Supplementary information [file 41598_2017_16804_MOESM1_ESM.pdf]

Supplementary information

Access to a main alphaherpesvirus receptor, located basolaterally in the respiratory epithelium, is masked by intercellular junctions

Jolien Van Cleemput, Katrien C.K. Poelaert, Kathlyn Laval, Roger Maes, Gisela S. Hussey, Wim Van den Broeck, Hans J. Nauwynck.

Supplementary experimental procedures

EHV1 purification and Dio-labelling

Culture fluids of EHV1-infected RK-13 cells were clarified by centrifugation at 60,000g for 2h at 4°C. The virus pellet was pooled onto a discontinuous OptiPrep™ gradient (Sigma-Aldrich, St. Louis, MO, USA) containing 10-30% (w/v) of iodixanol and centrifuged at 100,000g for 2.5h at 4°C. After centrifugation, purified opalescent virus bands were harvested at the interface of the 15% and 20% layers. To ensure efficient virus lipophilic labelling, the buffer was exchanged to HNE buffer (5 mM HEPES, 150 mM NaCl, 0.1 mM EDTA, pH 7.4) by the use of a 50K filter device (Millipore corporation, Bedford, MA, USA). While vortexing, 2 nM of 3,3'-Dioctadecyloxacarbocyanine perchlorate (Dio) dissolved in DMSO (Molecular probes, Oregon, USA) was added to the virus. Subsequently, unbound Dio was removed by centrifugation onto a MicroSpin™ G-50 fine column (GE Healthcare, Buckinghamshire, UK). The degree of Dio-labelled virus purity (>90%) was evaluated by simultaneous immunofluorescent staining of EHV1 gB with mouse monoclonal antibody 3F6 (kindly provided by Prof. U. Balasuriya, University of Kentucky, USA) and quantitative analysis by confocal microscopy.

Tissue collection and processing

Respiratory mucosal explant isolation and cultivation

The respiratory mucosa was stripped from the underlying cartilage and washed in PBS to remove excess blood. Tissues were cut into small square pieces (25 mm^2), placed with the epithelial side facing upwards onto fine-meshed gauzes and cultured in a 37°C, 5% CO_2, humidified incubator for 24h at air-liquid interface in serum-free medium containing DMEM/RPMI (Invitrogen, Paisley, UK), supplemented with 0.1 mg/mL gentamicin, 100 U/mL penicillin, 0.1 mg/mL streptomycin, and 0.25 µg/mL amphotericin B.

EREC isolation and cultivation

Tracheae were trimmed upon arrival in the lab and washed in PBS to remove excess blood. Tissues were submerged into an enzyme mix of 1.4% pronase (Roche Diagnostics Corporation, Basel, Switzerland) and 0.1% deoxyribonuclease I (Sigma-Aldrich) in calcium- and magnesium-free PBS supplemented with 0.45% glucose (VWR International, Leuven, Belgium), 1% sodium pyruvate (Invitrogen), 100 U/mL penicillin and 0.1 mg/mL streptomycin for 48h at 4°C. Detached cells were then incubated in DMEM/F12 (Invitrogen), containing 1% MEM non-essential amino-acids (Invitrogen), 2.4 µg/mL insulin (Sigma-Aldrich), 100 U/mL penicillin and 0.1 mg/mL streptomycin in a plastic petri dish for 2h to reduce fibroblast contamination by adherence. Isolated EREC were either seeded immediately or stored in liquid nitrogen at a density of $2 \cdot 10^6$ cells per cryovial until further use. EREC were seeded at a concentration of $1.8 \cdot 10^6$ cells/insert overnight into type IV collagen-coated (Sigma-Aldrich) 0.4µm pore size transwell cell culture wells (Costar, Corning, Fisher Scientific, Fair Lawn, USA) in DMEM/F12 (Invitrogen), supplemented with 5% non-heat inactivated FCS (Invitrogen), 1% MEM non-essential amino-acids, 100 U/mL penicillin, 1 mg/mL streptomycin, and 1.25 µg/mL amphotericin B. The next day, seeding medium was removed and the bottom platewells were filled with DMEM/F12, containing 2% Ultroser G (Pall Life Sciences; Pall Corp., Cergy, France), 100 U/mL penicillin, 0.1 mg/mL streptomycin, and 1.25 µg/mL amphotericin B (EREC medium). The transwell, comprising the apical surface of the EREC, was left empty to mimic an air-liquid interface. EREC were incubated in a 37°C, 5% CO_2 humidified incubator and medium was changed every 1-2 days until full differentiation. After 5-7 days, the EREC attained a trans-epithelial electrical resistance (TEER) of ~500-700 $\Omega \cdot cm^{-2}$. TEER was measured using an epithelial voltohmmeter (Millipore). The net resistance was calculated by subtracting the background resistance and multiplying the resistance by the surface area of the membrane.

Disruption of intercellular bridges of respiratory mucosal explants

First, 24-well culture dishes were filled with 1 mL of a solution containing 50% sterile 3% agarose (low temperature gelling; Sigma-Aldrich) and 50% 2X MEM (Invitrogen). Explants were placed onto the solidified agarose with the epithelial surface facing upwards. Additional agarose was added until the lateral surfaces of the mucosa were fully occluded. Explants were then exposed for 1h at 37°C to different drugs (8 mM EGTA, 500 mM NAC, 20 mM DTT or 50 mM β-mercaptoethanol in PBS). PBS supplemented with calcium and magnesium was used as a control. Finally, explants were washed 3 times to remove excess drugs and were fixed in phosphate-buffered 3.5% formaldehyde solution, either immediately or after an additional 24h incubation. An automated system was used for paraffin embedding of the samples (Thermo Scientific™ STP 120 Spin Tissue Processor). Eight μm paraffin sections were first deparaffinised in xylene, then rehydrated in descending grades of alcohol, subsequently stained with haematoxylin-eosin, dehydrated in ascending grades of alcohol and xylene and finally mounted with DPX (Sigma-Aldrich). Ten pictures on five different sections per treated explant were taken with an Olympus IX50 light microscope fitted with 40X objective. The percentage of intercellular space in the epithelium was measured using ImageJ software (ImageJ, U.S. National Institutes of Health, Bethesda, Maryland, USA). The region of interest (ROI, i.e. the epithelium) was drawn manually for each picture in the "ROI manager tool". Next, the threshold value to distinguish blank spaces from cellular material was determined and the percentage of blank spaces between the cells (i.e. the intercellular space) was calculated.

Immunofluorescent staining and confocal microscopy

Respiratory mucosal explants

Sixteen μm thick cryosections were cut using a cryostat at -20°C and loaded onto 3-aminopropyltriethoxysilane-coated (Sigma-Aldrich) glass slides. Slides were then fixed in 4% paraformaldehyde for 15min and subsequently permeabilized in 0.1% Triton-X 100 diluted in

PBS. Non-specific binding sites were blocked by 15min incubation with avidin and biotin (Invitrogen) at 37°C. To label late viral glycoproteins, a polyclonal biotinylated horse anti-EHV1 was used for 1h at 37°C[1], followed by incubation with streptavidin-FITC® (Invitrogen) for 1h at 37°C. The basement membrane of the tissues was stained with monoclonal mouse anti-collagen VII antibodies (Sigma-Aldrich), followed by secondary Texas Red® labelled goat anti-mouse antibodies (Invitrogen). Nuclei were detected by staining with Hoechst 33342 (Invitrogen). Slides were mounted with glycerol-DABCO and analysed using a Leica (TCS SPE) confocal microscope. The total number of plaques was counted on 50 cryosections and plaque latitude was measured using the Leica confocal software package. Five cryosections per explant were completely photographed and the percentage of infection in the epithelium (i.e. ROI) was determined using Image J software. The ROI (i.e. the epithelium) was drawn manually for each picture in the "ROI manager tool". Next, the threshold value to distinguish the FITC positive signal from the background signal was determined and the percentage of FITC positive signal (i.e. infection) was calculated.

Cellular glycosaminoglycans, heparan sulfate, chondroitin sulfate A and B and sialic acids were respectively stained with a monoclonal mouse anti-heparan sulfate antibody (10E4; Ambsio, Abingdon, UK), a monoclonal mouse anti chondroitin sulfate (CS-56; Bio-rad; Oxford; UK) or biotinylated Maackia Amurensis lectin (Vector Laboratories; Peterborough; UK) followed by a goat anti-mouse FITC® antibody or streptavidin-FITC®.

EREC

Antibodies were incubated directly in the transwells for 1h at 37°C. Cells were first incubated with a 1:1,000 dilution of a polyclonal rabbit anti-IEP antibody, kindly provided by Dr. D. O'Callaghan, Louisiana State University, USA. The diluent used was PBS containing 10% negative goat serum. This was followed by incubation with a goat anti-rabbit IgG FITC® conjugated antibody (Invitrogen). Nuclei were counterstained with Hoechst 33342 for 10min

at 37°C. Transwell membranes were excised from the culture inserts and mounted on glass slides using glycerol-DABCO. Slides were examined using a Leica confocal microscope. The total number of plaques was counted on 5 random fields of approximately $3 \cdot 10^4$ cells per insert. Plaque latitude was measured on 10 individual plaques using the Leica confocal software package.

Enzymatic removal of cell surface N-linked glycans and sialic acids prior to EHV1 inoculation

PNGase F (New England Biolabs, Ipswich, UK) removes complex, hybrid and oligomannose N-glycosylations and was applied onto apical or basolateral EREC surfaces for 12h at a concentration of 25,000 U/mL, diluted in EREC medium, supplemented with 10% glycobuffer (New England Biolabs; Ipswich; UK). Neuraminidase from *Vibrio cholera* (Sigma-Aldrich) has a broad substrate spectrum for sialic acids and was used for 1h at 50 mU/mL in PBS. The influenza A/Equine/Kentucky/98 (H3N8) strain served as a positive control during neuraminidase treatment of EREC. The virus was propagated in embryonated eggs and titrated onto Madin-Darby canine kidney (MDCK) cells. Influenza A strains can easily infect MDCK cells through interaction with cell-associated sialic acids, which can be removed with neuraminidase[2]. Equine influenza virus (EIV) A nucleoprotein was stained with the mouse monoclonal antibody HB-65 (ATCC) and subsequently visualized with FITC®-labelled secondary goat anti-mouse IgG antibodies. Nuclei were counterstained with Hoechst 33342 and coverslips were mounted using glycerol-DABCO. The percentage of positive cells was calculated based on the total number of positive cells out of 300 randomly selected cells.

Correct cleavage of the respective sialic acids was corroborated by immunofluorescent staining of EREC with biotinylated Maackia Amurensis Lectin II (Vector laboratories). The complex was subsequently stained with Streptavidin-FITC® (Invitrogen). Ten z-stack confocal pictures were taken to distinguish apical from basolateral treatment. The means of the fluorescent apical

or basolateral signals were compared with Image J software and dropped significantly after treatment with neuraminidase, compared to control. Following enzymatic treatment, cells were washed 3 times with DMEM/F12 and the inoculum was delivered on top of the respective surfaces for 1h at 37°C. Concurrently, and as a positive control for enzymatic treatment, MDCK cells were treated with neuraminidase before inoculation with EIV. After 1h inoculation, unbound virus particles were removed by washing and cells were incubated for 10 hours before fixation in methanol, as described above.

Supplementary figures

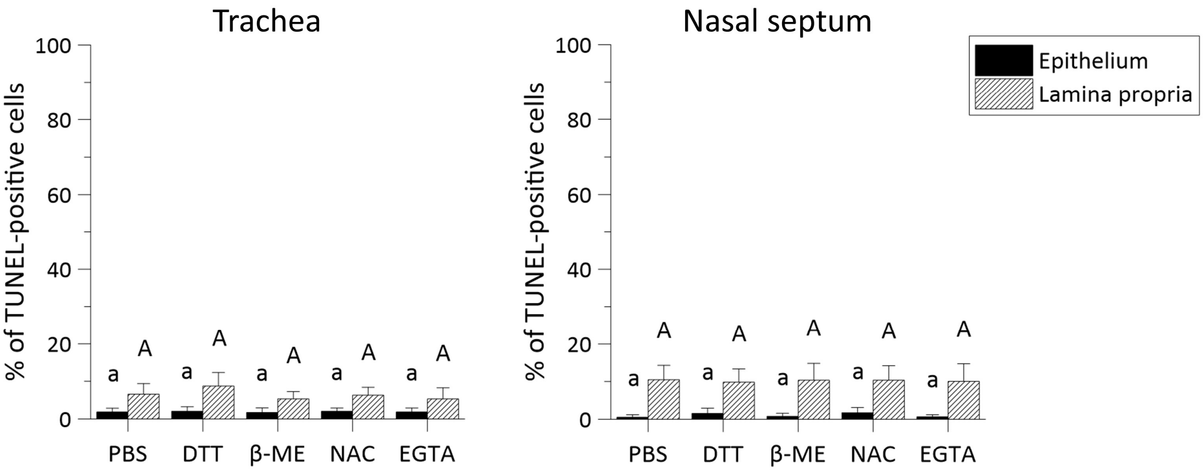

Figure S1. Cell viability in respiratory mucosal explants

TUNEL-staining data of tracheal ME (left) and nasal ME (right) after different treatments. Three independent experiments were performed and data are represented as means + SD. The lower case letters indicate significant (P<0.05) differences in the epithelium, while the upper case letters indicate significant differences in the lamina propria.

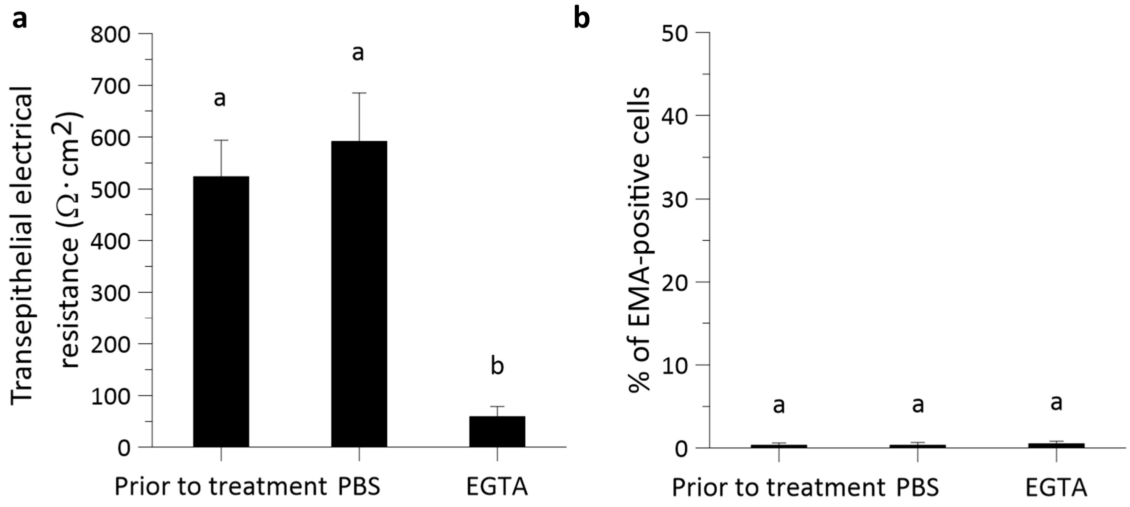

Figure S2. The disruption of intercellular bridges in EREC

(a) Trans-epithelial electrical resistance of EREC prior to treatment and after 30min treatment with PBS (control) or EGTA. Three independent experiments were performed and the data are represented as means + SD. Different letters indicate significant ($P<0.05$) differences. (b) EMA-staining confirmed no significant ($P<0.05$) differences in cell viability after different treatments, when compared to cell viability prior to treatment. Three independent experiments were performed and data are represented as means + SD. Different letters indicate significant ($P<0.05$) differences.

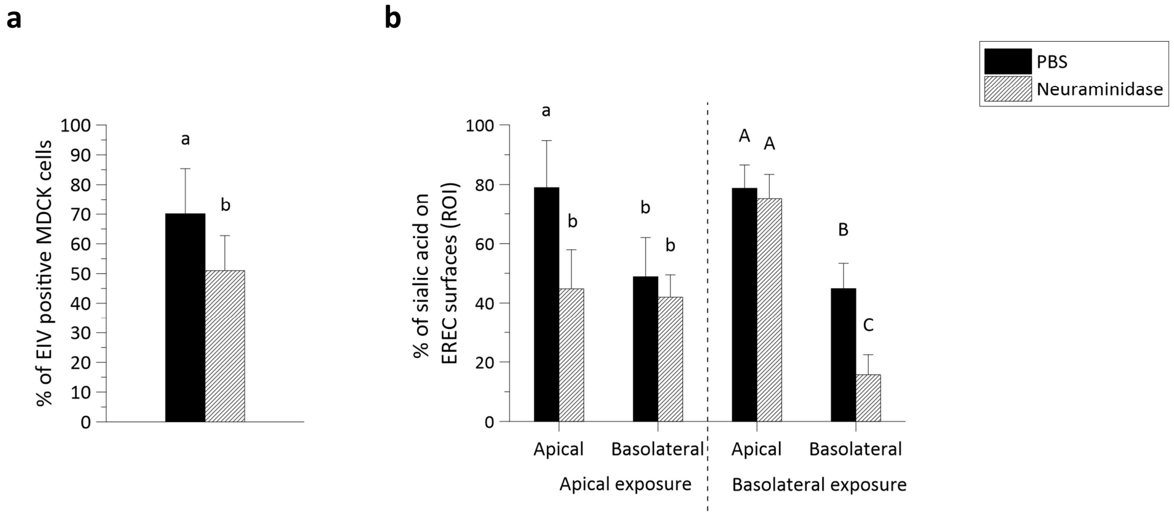

Figure S3. Validation neuraminidase assay

(a) During enzymatic treatment, MDCK cells were similarly pre-treated with neuraminidase before inoculation with EIV. Cells were fixed at 6hpi in methanol and EIV-positive cells were visualized using the monoclonal HB65 antibody. Five random fields of approximately 100 cells were screened to calculate the percentage of EIV-positive cells. Experiments were performed in triplicate. Data are represented as means + SD and different letters represent significant differences. (b) Correct sialic acid-cleavage from EREC surfaces was confirmed by confocal analysis of 10 different z-stacks per treatment. EREC were grown to confluency on transwells and treated at either the apical surface (left) or basolateral surface (right) with neuraminidase or control PBS for 1h at 37°C. Cells were then fixed in PFA and permeabilized in Triton X, sialic acids were stained with biotinylated Maackia Amurensis lectin and the percentage of fluorescent signal in either the apical or the basolateral domain of EREC was calculated. Data are represented as mean + SD. Significant differences after apical exposure are indicated by different lower case letters and after basolateral exposure by different upper case letters.

Supplementary references

1 van der Meulen, K., Vercauteren, G., Nauwynck, H. & Pensaert, M. A local epidemic of equine herpesvirus 1-induced neurological disorders in Belgium. *Vlaams Diergeneeskundig Tijdschrift* **72**, 366-372 (2003).

2 Stray, S. J., Cummings, R. D. & Air, G. M. Influenza virus infection of desialylated cells. *Glycobiology* **10**, 649-658 (2000).
